# The salience of nonlinearities in the boreal winter response to ENSO: Arctic stratosphere and Europe

**DOI:** 10.1007/s00382-019-04805-1

**Published:** 2019-05-24

**Authors:** Israel Weinberger, Chaim I. Garfinkel, Ian P. White, Luke D. Oman

**Affiliations:** 1 The Fredy and Nadine Herrmann Institute of Earth Sciences, Hebrew University of Jerusalem, Jerusalem, Israel; 2 NASA Goddard Space Flight Center, Greenbelt, MD USA

## Abstract

The Arctic stratospheric response to El Niño (EN) and La Niña (LN) is evaluated in a 41 member ensemble of the period 1980 to 2009 in the Goddard Earth Observing System Chemistry-Climate Model. We consider whether the responses to EN and LN are equal in magnitude and opposite in sign, whether the responses to moderate and extreme events are proportionate, and if the response depends on whether sea surface temperature anomalies (SSTs) peak in the Eastern Pacific (EP) or Central Pacific (CP). There is no indication of any nonlinearities between EN and LN, though in ~ 15% of the ensemble members the stratospheric sudden warming (SSW) frequencies for EN and LN are similar, suggesting that a similar SSW frequency for EN and LN, as has occurred over the past ~ 60 years, can occur by chance. The response to extreme EN events is not proportionate to the amplitude of the underlying SST anomalies in spring. EP EN events preferentially increase zonal wavenumber 1 and decrease zonal wavenumber 2 as compared to CP EN events, however the zonal-mean Arctic stratospheric and subpolar surface response is generally little different between EP EN and CP EN once one accounts for the relative weakness of CP events. These differences between EP and CP events and between moderate and extreme EN events only emerge if at least 25 events are composited, however, due to the small signal-to-noise ratio, and hence these differences may be of little practical benefit.

## Introduction

El Niño Southern Oscillation (ENSO), i.e. the warm (El Niño hereafter EN) and cold (La Niña hereafter LN) phases of the equatorial Pacific coupled atmosphere-ocean phenomenon, impacts the global atmospheric circulation in both the troposphere (Horel and Wallace 1981; Ropelewski and Halpert 1987; Halpert and Ropelewski 1992; Trenberth et al. 1998) and stratosphere (van Loon and Labitzke 1987; Hamilton 1993; Domeisen et al. 2019). EN leads to an accelerated Brewer–Dobson circulation and a warmer polar stratosphere on average by several degrees Kelvin (Sassi et al. 2004; Garcia-Herrera et al. 2006; Garfinkel and Hartmann 2007; Camp and Tung 2007; Free and Seidel 2009).

The Arctic stratospheric response during EN events is driven specifically by the deepened Aleutian low (e.g. Barnston and Livezey 1987), which constructively interferes with the climatological stationary wave pattern and leads to a strengthened wave flux into the stratosphere (i.e. linear interference; Garfinkel and Hartmann 2008; Garfinkel et al. 2010; Smith et al. 2010; Smith and Kushner 2012). Episodes of prolonged upward wave flux can lead to sudden stratospheric warming (SSW) events (Polvani and Waugh 2004; Sjoberg and Birner 2012), and on average, modulate surface climate (Cagnazzo et al. 2009; Ineson and Scaife 2009; Bell et al. 2009) and thus increase weather predictability (Sigmond et al. 2013) in the Euro-Atlantic region for weeks.

It is not clear to what extent the Arctic stratospheric response to LN is opposite to that of EN. While in the seasonal mean strong LN events appear to lead to a stronger and colder than normal Arctic polar vortex (Garfinkel and Hartmann 2007; Iza et al. 2016), there is no reduction in the frequency of SSW events during LN winters (Butler and Polvani 2011; Polvani et al. 2017; Domeisen et al. 2019). Two explanations have been offered as to why LN should not lead to reduced SSW frequency: first, the North Pacific ridge associated with LN does not reach the subpolar Northwest Pacific where it could most efficiently destructively interfere with the climatological stationary waves (Garfinkel et al. 2012), and second, the North Pacific ridge and enhanced blocking associated with LN extends over the Northeast Pacific where it can lead to enhanced wavenumber 2 driving of the vortex (Barriopedro and Calvo 2014). However, models do not succeed in capturing such a relationship between LN and SSW (Taguchi and Hartmann 2006; Bell et al. 2009; Garfinkel et al. 2012; Li and Lau 2013; Domeisen et al. 2015; Polvani et al. 2017; Song and Son 2018), and hence it is unclear whether the lack of a reduced SSW frequency in LN in the reanalysis record might simply be related to random variability in a relatively short record, or alternately whether models have biased teleconnections during LN (Butler and Polvani 2011; Garfinkel et al. 2012; L’Heureux et al. 2017). Furthermore, the observed LN-SSW relationship has also been shown to be sensitive to how LN events and SSW events are classified (Polvani et al. 2017; Song and Son 2018).

No two EN events have identical sea surface temperature (SST) anomalies, and it is unclear whether differences in the SST pattern are crucial for the stratospheric response. EN events differ both in their magnitude, with some events—e.g. 1997/1998, 1982/1983 and 2015/2016—stronger than others, and in the location within the tropical Pacific where maximum SST anomalies are observed. Specifically, for Central Pacific (CP) EN events, also called a dateline EN event (Larkin and Harrison 2005), SST anomalies peak in the central equatorial Pacific, while for East Pacific (EP) EN events SST anomalies peak in the eastern equatorial Pacific.

While it is reasonable to expect that stronger events lead to stronger tropical precipitation anomalies, the increase is not linear (Frauen et al. 2014; Rao and Ren 2016a). These nonlinearities could lead to a nonlinear stratospheric response, but detecting such a nonlinearity in observations is difficult. Rao and Ren (2016a) composite moderate EN and strong EN events separately and conclude that moderate events modulate the northern winter stratosphere more robustly. However, the salience of such nonlinearities appears to depend on the methodology adopted as it is difficult to detect such a nonlinearity given the large amount of internal variability in Figure 11 of Rao and Ren (2016a). Modeling studies disagree as to whether nonlinearities are present: Rao and Ren (2016b) find evidence for nonlinearity, but Richter et al. (2015) find that the modeled response to the 1997/1998 and 1982/1983 EN events is more than double the response to moderate events, implying that the response to EN is linear. The recent study by Zhou et al. (2018) argues that the response in spring is linear, and any nonlinearities are confined to earlier in winter. The European sector response may be similarly nonlinear (Toniazzo and Scaife 2006; Bell et al. 2009).

Finally, both EP EN and CP EN events lead to a deepened Aleutian low (Yu and Kim 2011) though the deepening is stronger for eastern Pacific events especially in early winter (Yu and Kim 2011; Sung et al. 2014). CP EN events lead to a southward and westward displacement of the anomalously low sea level pressure relative to EP EN events (Yu and Kim 2011; Garfinkel et al. 2013, 2018b), and this southward displacement of the Aleutian low, as well as the relative weakness of the amplification, could lead to weaker impacts in the Arctic stratosphere during CP EN events. However, it is not yet possible to discriminate between the teleconnections of CP EN and EP EN events in a statistically robust manner that is not sensitive to methodological choices in observations (Garfinkel et al. 2013; Iza and Calvo 2015). Modeling studies have also not yet reached a consensus on whether robust differences exist between the stratospheric response to CP EN and EP EN events. Some studies have concluded that only EP EN events lead to a weakening of the vortex (Xie et al. 2012; Calvo et al. 2017), some find little robust difference in the response between CP EN and EP EN events (Hegyi et al. 2014; Hurwitz et al. 2014), while others argue that both lead to weakening of the polar vortex, but with the vortex weakening during EP EN more pronounced in early winter (Garfinkel et al. 2013) or in the absence of SSWs (Iza and Calvo 2015). Even if the modulation of the vortex is somewhat weaker during CP EN events, the impacts in the Euro-Atlantic sector found in some studies is stronger (e.g. Graf and Zanchettin 2012; Garfinkel et al. 2013), suggesting that EN can modulate the vortex via a purely tropospheric route (Butler et al. 2014; Rodríguez-Fonseca et al. 2016; Jiménez-Esteve and Domeisen 2018).

Given the importance of the stratosphere for European sector winter variability (Charlton et al. 2003; Baldwin et al. 2003), and given the uncertainties in the European response to ENSO (Toniazzo and Scaife 2006; Brönnimann 2007), here we seek to clarify some of these lingering uncertainties in the linearity of the stratospheric response to ENSO. We address these lingering uncertainties by first identifying the nonlinear responses to ENSO in the wavedriving of the Arctic polar vortex, in the Arctic stratosphere, and in the subsequent downward impact on the troposphere. For each of these regions, We address three target questions:

1. Are the responses to EN and LN equal in magnitude and opposite in sign?
2. Is the magnitude of the response to extreme EN events proportionately stronger than that of moderate EN events?
3. Are the responses to different EN flavors (i.e, EP EN and CP EN events) similar?

In the rest of this paper we refer to the atmospheric response as ‘linear’ when the responses to EN and LN are equal in magnitude and opposite in sign, the response to moderate vs extreme events is proportional and there is no significant difference between the responses to EP EN and CP EN events. Whenever nonlinearities are found, we ask the additional question: how many samples (events) does one need in order to establish the robustness of these nonlinearities?

After introducing the data and methods in Sects. 2 and 3, we demonstrate that the modulation of the wave-driving of the vortex by ENSO is nearly linear, though there are differences in the wavenumber composition of the modulated wave-driving between CP EN and EP EN events (Sect. 4) that in turn affects the morphology of stratospheric sudden warmings for each El Niño flavor. The Arctic stratospheric response to ENSO is effectively linear in winter, though in spring extreme EN events do not lead to a proportionately stronger response (Sect. 5). The linearities and nonlinearities of the stratospheric response are mostly mirrored in the surface response (Sect. 6). However detecting these nonlinearities is very difficult: the anomalies forced by ENSO are small as compared to the internal variability in the extratropical atmosphere, and specifically, individual ensemble members may display apparent nonlinearities in SSW frequency that are in many ways similar to those observed even if none exist in the ensemble mean response (Sect. 7).

## Data

The foundation of this study is an ensemble of integrations conducted using the Goddard Earth Observing System Chemistry-Climate Model (GEOSCCM Rienecker et al. 2008; Oman and Douglass 2014) described in Garfinkel et al. (2018a, b). This model couples the GEOS-5 (Rienecker et al. 2008; Molod et al. 2012) atmospheric general circulation model to the comprehensive stratospheric chemistry module StratChem (Pawson et al. 2008). The model has 72 levels, with a model top at 0.01 hPa, and the horizontal resolution of all integrations discussed here is 2$^\circ$ latitude $\times$ 2.5$^\circ$ longitude. The model spontaneously generates a Quasi-Biennial Oscillation (QBO) (Molod et al. 2012), though the QBO phase is not synchronized with the observed QBO or among the experiments. One of the integrations of Garfinkel et al. (2018a) ends in December 2008, and we do not include this integration here. 41 ensemble members covering the period 1980 to 2009 are analyzed, and all are free-running and have observed sea surface temperature (SST) variations imposed globally. Full details of the model ensemble are included in Garfinkel et al. (2018a). These same experiments were analyzed in Garfinkel et al. (2018a, b) with a focus on the surface impacts of ENSO over North Pacific and North America and on the tropical stratosphere; here we focus on the Arctic stratospheric response and the subpolar surface response.

An imposed-SST ensemble like the one analyzed here allows a natural comparison to the observed response to ENSO, and model output is compared to meteorological fields from MERRA (Modern-era retrospective analysis for research and applications; Rienecker et al. 2011) and ERA-Interim (ERAI) reanalysis (Dee et al. 2011).

## Methods

The ENSO phase for each winter season is defined by SST anomalies in the Niño3.4 region (5S–5N, 170W–120W) in version 5 of the ERSST dataset (Huang et al. 2017) with a 1980–2009 base period. We first apply a 1–2–1 running mean smoothing on the Niño3.4 index for each set of three months (e.g. weights of 1/4 are applied to months $\mathrm{n}+1$ and $\mathrm{n}-1$ and a weight of 1/2 is applied to month n). We then define a season as EN or LN based on the NDJF seasonal mean Niño3.4 anomalies. Other studies have adopted alternate definitions, and in the appendix we discuss sensitivity to the detailed manner in which events are chosen. In the interest of simplicity the main text adopts the NDJF seasonal mean definition.

EN events are identified when SST anomalies in the Niño3.4 region, are larger than 0.5 K. EN events are further categorized as Eastern Pacific El Niño (EP EN) and Central Pacific El Niño (CP EN). EP EN events are identified when the Niño3 region (5S–5N, 210E–270E) SST anomaly is positive and 0.1 K larger than the Niño4 region (5S–5N, 160E–210E) anomaly (similar to Hurwitz et al. 2014), after applying a 1–2–1 running mean smoothing to the Niño3 and Niño4 indices. As we would like to discern whether differences in the response to EP EN and CP EN are due to differences in the position of tropical convection and not due to differences in event amplitude, we further divide the EP EN events into “extreme” events (i.e. 82/83 and 97/98) and “moderate” events (1986/1987, 1991/1992, See Table 1). CP EN events are identified when the Niño4 SST anomaly is positive and 0.1 K larger than the Niño3 SST anomaly, after applying a 1–2–1 running mean smoothing to the Niño3 and Niño4 indices. Composited anomalies during EP and CP events depend on the specific definition adopted, however the two years identified herein as CP EN (1994/1995 and 2004/2005) are so classified for nearly all CP definitions (e.g Garfinkel et al. 2013; Johnson and Kosaka 2016). The 1991/1992 event can be classified as a Modoki event (Ashok et al. 2007; Garfinkel et al. 2013), however this classification is mainly due to the cold SST anomalies that were present over the far Western Pacific as SSTs in the East Pacific Niño3 region were indeed warmer than those in the Central Pacific Niño4 region. Note that SST anomalies in the Niño3.4 region are still $\sim 30\%$ stronger for the moderate EP EN events than the CP EN events. All remaining EN years, in which the Niño3 and Niño4 anomalies are within 0.1 K, are categorized as “other EN events”.

**Table 1:** Events included for each ENSO composite

ENSO composites	
Composite	Years
Moderate EP EN	1986/1987, 1991/1992
Extreme EP EN	1982/1983, 1997/1998
CP EN	1994/1995, 2004/2005
Other EN	1987/1988, 2002/2003, 2006/2007
EP LN	1984/1985, 1995/1996, 1999/2000, 2005/2006, 2007/2008
CP LN	1983/1984, 1988/1989, 1998/1999, 2000/2001, 2008/2009

LN events are identified when SST anomalies in the Niño3.4 region are below $-0.5$ K. In our presentation of the response to LN, we do not separately consider CP LN and EP LN events, because the response to CP LN and EP LN events was found to not be robustly different in our ensemble for any metric despite the availability of hundreds of model-seasons (not shown). We therefore form a single LN composite, and we also include in this LN composite years that cannot be unambiguously classified as either EP or CP.

The years included in each composite are listed in Table 1. For figures which compare the emergence of nonlinearity in the composited response to moderate EP EN events as compared to extreme EP EN events, we weight the response in the extreme EP EN composite by its underlying Niño3.4 SST anomaly.

Most ENSO events peak in the early winter or late fall, and decay through the following spring. However, the lower-stratospheric response in observations (Manzini et al. 2006; Garcia-Herrera et al. 2006), in previous modeling studies (e.g. Cagnazzo et al. 2009), and in the model experiments described in this paper peaks in late winter, and hence we consider the response separately from December through February and for early spring (March and April).

Sudden stratospheric warming (SSW) events are defined according to the zonal wind reversals at 10 hPa, 60N following Charlton and Polvani (2007) and its corrigendum, and the corresponding observed events are as listed in Butler et al. (2014); we also explore sensitivity to using 10 hPa, 65N zonal wind. For the observational SSW frequency we consider the extended period from 1958 to 2013. When considering the frequency of SSW during alternate phases of ENSO we include the reanalysis results of Polvani et al. (2017) who used the NOAA Climate Prediction Center (CPC) definition to identify ENSO events based on the ERSST version 4 dataset (see the Appendix). Split and displacement SSWs are computed as in Seviour et al. (2013), and the specific application of this algorithm to these GEOSCCM integrations is described in White et al. (2019).

Monthly anomalies are computed as follows. A monthly climatology over the full duration of each model experiment, reanalysis product, and observational dataset is computed, and is then subtracted from the raw fields to generate monthly anomalies. All anomalies are then detrended by removing the linear trend over the course of the simulated period. Daily anomalies are computed analogously, except that a daily climatology is used.

When considering differences between ENSO flavors and phases, we utilize two methodologies, and in all cases the null hypothesis is that the response to ENSO is linear. The first is a compositing approach, and statistical significance for the anomalies in a composite relative to climatology and for the difference between two composites is computed using a two-tailed Student t test, unless otherwise specified. The compositing approach is applied to all three target questions.

The second is a regression approach, and we apply it to target questions 1 and 2 only. The SST anomalies in the Niño3.4 region during NDJF are used as a predictor for the extratropical response. We consider whether the response to LN is equal and opposite to that of EN by first computing the interaction term when both LN and moderate EN events are included in the same regression analysis (p 220–228 of McDonald 2014). If the slopes are found to be significantly different then regression lines for moderate EN and LN are plotted separately, however in practice the slopes are statistically indistinguishable for all figures in this paper. We therefore plot a single gray regression line for moderate EN and LN events. All LN events in this period were weaker than either extreme EN event, and so we only compare LN to moderate EN events. Statistical significance of the slope of the regression line is computed using a two-tailed Student t test.

We also consider whether the impact of extreme EN events is proportionately stronger than the response to moderate EN events by computing a linear best-fit and a polynomial best fit (e.g. $$\hbox {T}_{\mathrm{85 hPa, pole}}$$$$\sim a \times EN^2+b \times EN$$) for all EN events, and then comparing the $$R^2$$ of the linear best-fit to the adjusted $$R^2$$ (Eq. 3.30 of Chatterjee and Hadi 2012) of the polynomial best fit. The adjusted $$R^2$$ takes into account the likelihood that a polynomial predictor will reduce the residuals by unphysically over-fitting the data. If the adjusted $$R^2$$ for the polynomial fit is less than the linear $$R^2$$, then a linear best-fit more succinctly describes EN’s teleconnection.

Our results will highlight the ability of the large internal variability in the atmosphere to mask the response to ENSO. This large internal variability also makes it difficult to evaluate whether the model response to ENSO is realistic (Deser et al. 2017). However a necessary prerequisite for comparing observed and modeled ENSO teleconnections is for the model to realistically simulate a similar amount of *variance* as compared to that observed, as otherwise the model does not satisfactorily capture internal atmospheric variability (Deser et al. 2017). We therefore assess whether GEOSCCM simulates a realistic amount of variance for each metric considered in this paper in Appendix B.

## Changes in wave-driving

**Fig. 1:**
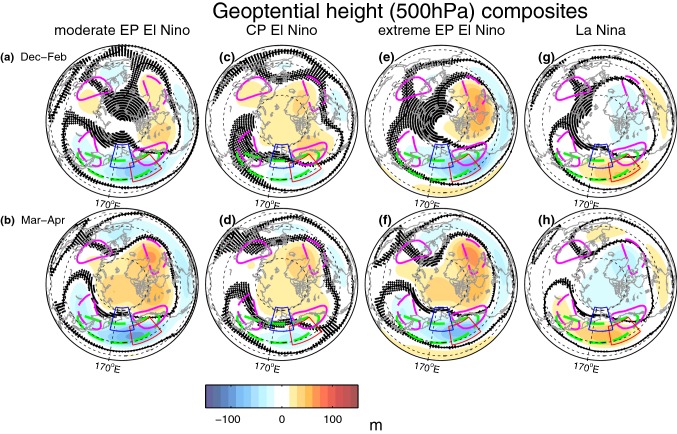
Geopotential height at 500 hPa response to ENSO in GEOSCCM. The contour interval is 15 m. A red box (from 35N–55N, 190E–220E) demarcates the region in which the North Pacific response to ENSO peaks (as discussed in Garfinkel et al. (2018b)), while the blue box (from 52.5N–72.5N, 165E–195E) demarcates the precursor region from Garfinkel et al. (2012) (see also Fig. 10). **a**, **b** moderate EP EN events: 1986/1987, 1991/1992; **c**, **d** CP EN events; **e**, **f** extreme EP EN: 1982/1983 and 1997/1998; **g**, **h** LN. (top) December through February and (bottom) March and April. The troughs and ridges of the 50 m contour climatological wavenumber-2 pattern are shown in magenta dashed and solid lines respectively, while the 50 m contour of the climatological wavenumber-1 trough in the Pacific sector is shown in a dashed green line. Statistical significance is computed using a two-tailed Student’s t test with a 95% confidence threshold using all 41 ensemble members, and stippling indicates grid boxes that are not significant using a false discovery rate of 10% following Wilks (2016)

We begin with composites of 500 hPa geopotential height using the full ensemble for moderate EP EN, CP EN, extreme EP EN (97/98 and 82/83), and LN (Fig. 1). A comparable figure but for sea level pressure is included in Garfinkel et al. (2018b). All three EN composites show the canonical wavetrain pattern in the Western Hemisphere, with a low in the Northeastern Pacific, a high over Canada, and a low near the Eastern United States. Anomalies in the LN composite are nearly opposite to those in the EN composites. Each panel in Fig. 1 includes the 50 m contour of the climatological zonal wavenumber-1 (in green) and wavenumber-2 (in magenta) eddy height field in GEOSCCM. This field can be compared to the observed eddy height field in figure 2 in Garfinkel et al. (2010). GEOSCCM simulates realistic climatological stationary waves, though wave-2 is too weak: its amplitude at 50N at 500 hPa is 75.3 m in GEOSCCM as compared to 87.5 m in MERRA.

**Fig. 2:**
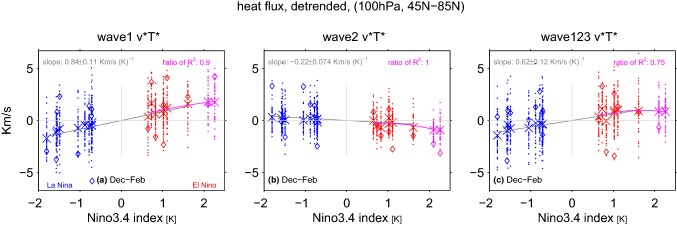
100 hPa heat flux response to ENSO in winter (December through February). **a** Anomalies in zonal wavenumber-1 heat flux area-weighted from 45N to 85N stratified by SST anomalies in the Niño3.4 region, after the component of the variance linearly associated with the QBO at 50 hPa and a linear trend has been removed; **b** as in (**a**) but for zonal wavenumber 2; **c** as in (**a**) but for the sum of wavenumbers 1,2, and 3. LN winters are in blue, moderate EN winters (whether EP, CP, or marginal) are in red, and extreme EN winters are in magenta. A linear least-squares best fit is shown in each panel, and the slope is indicated. If the slope of the best fit is statistically significantly different for LN as compared to moderate EN, we show the slopes separately; if not, then a single slope and a single best-fit line is included. An additional polynomial best-fit is shown considering both moderate and extreme EN events in magenta. The ratio of the adjusted $R^2$ for the polynomial best-fit as compared to the $R^2$ for the linear best-fit is indicated in magenta. The ensemble mean response is indicated with a large x, and each ensemble member with a dot. The response in MERRA reanalysis is shown with a diamond

We first focus on the modulation of wave-1 by ENSO. As discussed in Garfinkel and Hartmann (2008) and Ineson and Scaife (2009), EN leads to low height anomalies over the North Pacific of the same sign as, and hence that constructively interfere with, the climatological stationary wave-1. This effect is qualitatively similar for all three EN composites (Fig. 1). During LN, on the other hand, higher heights over the North Pacific lead to destructive interference (Ding et al. 2017). Previous work has linked these contrasting responses under EN and LN to opposite-signed anomalies in upward propagation of wave flux in the lower stratosphere (e.g. Garfinkel and Hartmann 2008). We diagnose changes in the upward wave flux into the lower stratosphere by the heat flux at 100 hPa in Fig. 2 (Andrews et al. 1987). Specifically Fig. 2a shows the wave-1 heat flux response at 100 hPa for all ensemble members and all non-neutral ENSO events. Each event is stratified by its SST anomaly, and we indicate the range of responses across all 41 ensemble members (each ensemble member is a dot), the response in the MERRA reanalysis (a diamond), and the ensemble mean (a large x). We then compute the linear best-fit regression line for moderate EN and LN, and if the difference in slope between the regression line for moderate EN and LN is statistically significant, we list the slope separately for each. If the slopes are statistically indistinguishable, then one slope is quoted and only one line added.

There is no evidence to reject the null hypothesis that LN and EN have equal and opposite effects on wave-1 heat flux, as the slope of the best-fit lines for each are statistically indistinguishable despite hundreds of years of model output. Similarly, the response to extreme EN events is proportionately stronger than the response to moderate EP EN events, as the adjusted $R^2$ of the polynomial fit is smaller than the $R^2$ of the linear fit ($R^2$ ratio is 0.9).

**Fig. 3:**
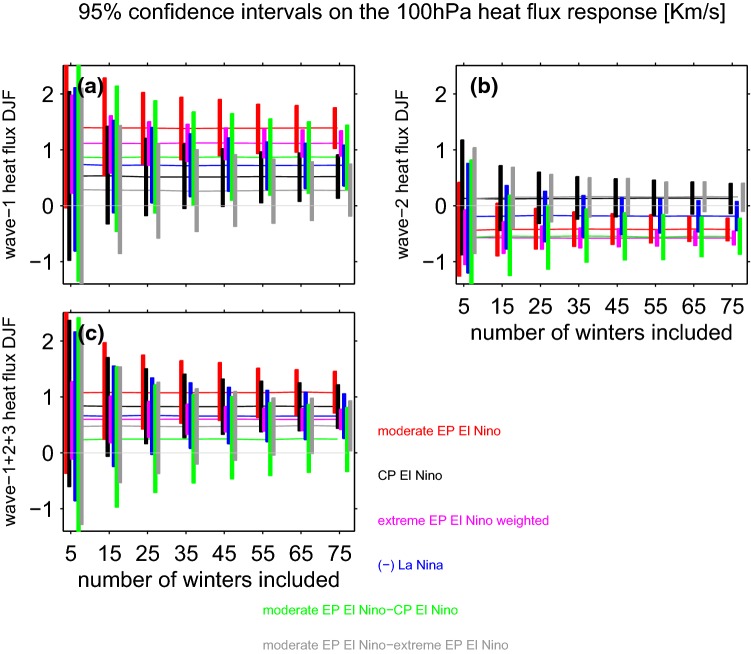
95% confidence intervals on the response to ENSO when the full ensemble is subsampled. Heat fluxes in December through February for **a** wave-1, **b** wave-2, **c** the sum of wave numbers 1, 2 and 3. The response to LN is multiplied by $-1$, and the magnitude of the response to extreme EP EN by the ratio of the magnitude of the Niño3.4 anomalies for the moderate EP EN composite and the extreme EP EN composite (which are $60\%$ stronger during extreme EP EN than for the two moderate EP EN events)

Do CP EN have a similar impact as EP EN events on wave-1? A compositing approach indicates that CP EN leads to significantly less wave-1 heat flux than moderate EP EN if we consider the entire 41-member ensemble by approximately a factor of two (Fig. 3a). Note that the North Pacific low is also weaker during CP EN (Fig. 1a as compared to Fig. 1c), and the tropical SST anomalies are weaker as well (Garfinkel et al. 2018b). How many CP EN events and EP EN events must be composited before the wave-1 heat flux in the lower stratosphere in winter becomes significantly different? To answer this question we introduce a bootstrapping methodology that will also be used in Sects. 5 and 6. We bootstrap with replacement the wave-1 heat flux response for a subsample of the full 41-member ensemble, with the size of the subsample increasing from 5 randomly selected events up to 75 randomly selected events for each ENSO composite. We create 2000 such bootstrapped subsamples for each subsample-size. We then compute the mean and the top and bottom 2.5% quantiles without making any assumption on the nature of the distribution, and hence form 95% confidence intervals of the response. This allows us to quantify how the uncertainty in the wave-1 response decreases as the number of events averaged together increases (Fig. 3a). The green line shows the difference between moderate EP EN and CP EN events, and the difference is significant when the green line does not touch the zero line. Approximately 35 individual events are necessary before the difference in wave-1 between moderate EP EN and CP EN events in Fig. 3a becomes statistically significant.

We next turn our attention to wave-2. The climatological wave-2 field is composed of a ridge over the West Coast of North America and a trough over Northeast Asia (Fig. 1), and the North Pacific trough in response to moderate EP EN and extreme EN destructively interferes with this North American ridge (consistent with e.g. Taguchi and Hartmann 2006; Garfinkel and Hartmann 2008). CP EN, in contrast, leads to constructive interference with this ridge (Fig. 1). As discussed in Garfinkel et al. (2018b), the North Pacific trough associated with CP EN is characterized by a higher zonal wavenumber due to the details of how CP EN modulates tropical convection, and hence the North Pacific trough does not reach the far-Northeastern Pacific. The net effect is that the Canadian ridge during CP EN overlies the climatological wave-2 ridge. Approximately 30 individual events are necessary before the difference in wave-2 between EP EN and CP EN becomes statistically significant (green lines on Fig. 3b). No other nonlinearities are evident for wave-2: the response to LN events is equal and opposite as compared to EN events (the slopes of the best-fit linear lines for LN and moderate EN are statistically indistinguishable), and the response to extreme EN events is somewhat more than proportionately stronger than the response to moderate EP EN events, though there is little statistical justification for preferring a polynomial fit as the adjusted $R^2$ of the polynomial fit is similar to the $R^2$ of the linear fit ($R^2$ ratio is 1.0).

The total planetary wave heat flux (defined here as wavenumbers 1 through 3) at 100 hPa is shown in Fig. 2c. The response to LN events is equal and opposite as compared to EN events of comparable magnitude. While the response to extreme EN events is not quite proportionately stronger than the response to moderate EP EN events, there is little statistical justification for preferring a polynomial fit given the adjusted $R^2$ of the polynomial fit. The location of the maximum SSTs also appear to lead to a slightly different response: as a consequence of the difference in wave-2, the total planetary wave heat flux is robustly different for EP EN as compared to CP EN events (Fig. 3c). Specifically, the enhanced wave-1 heat flux during both EP EN and CP EN events is partially compensated by reduced wave-2 for EP EN events, but dominates the net effect during CP EN events. As to the salience of this nonlinearity, the green lines on Fig. 3c show that there is no significant difference between the total heat flux response to moderate EP EN as compared to CP EN even if 75 events are considered. The gray line on Fig. 3c indicates the difference in planetary wave heat flux between moderate and extreme EP EN events, after the response for the extreme EP EN events has been weighted by the ratio of the Niño3.4 index for the two composites. A robust nonlinearity between moderate and extreme EP EN events can be identified when the gray line does not cross the zero-line. We find that $\sim 65$ moderate and extreme events must be composited before the relative weakness of heat flux for extreme EN events becomes robust.

In conclusion, the wavenumber composition of the upward flux is dependent on the flavor of the EN event. The responses to LN and EN are symmetric, and the response to extreme EN is essentially proportionately stronger than the response to moderate EN events, in our GEOSCCM ensemble. The implications in the stratosphere for these changes in the wave-driving are discussed in the next section.

It is important to note that in all panels of Fig. 2, there is substantial intra-ensemble variability. The winter stratosphere is characterized by unforced internal variability, and consistent with this more than 30 events are needed to identify differences in the response to moderate EP EN and CP EN in Fig. 3a, b. Hence the anomalies in a given winter can be opposite in sign to the forced response as deduced from the ensemble mean.

## Changes in the Arctic stratosphere

**Fig. 4:**
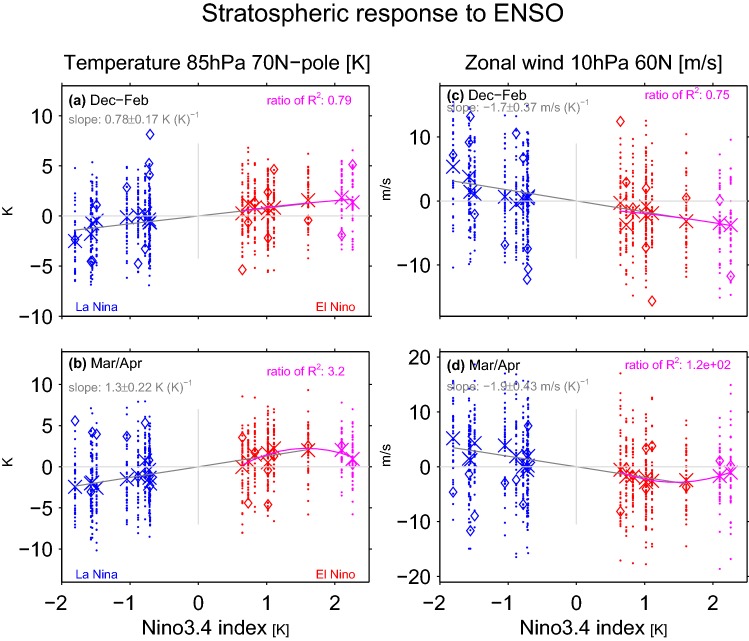
As in Fig. 2 but for the stratospheric response to ENSO in boreal winter and spring. **a**, **b** Anomalies in 85-hPa temperature area-weighted from 70N to the pole, after the component of the variance linearly associated with the QBO at 50 hPa has been removed; **c**, **d** as in **a**, **b** but for zonal mean zonal wind at 10 hPa and 60 N. (top) December, January, and February; (bottom) March and April

We now turn our attention to the linearity of the stratospheric response. Figure 4a, b consider the temperature response in the lower stratosphere during December through February and during March and April, respectively, and Figure 4c, d consider the zonal mean zonal wind response at 60N and 10 hPa. The responses to EN and LN are equal in magnitude and opposite in sign, as the slope of the linear best-fits for EN events and for LN events are statistically indistinguishable.

**Fig. 5:**
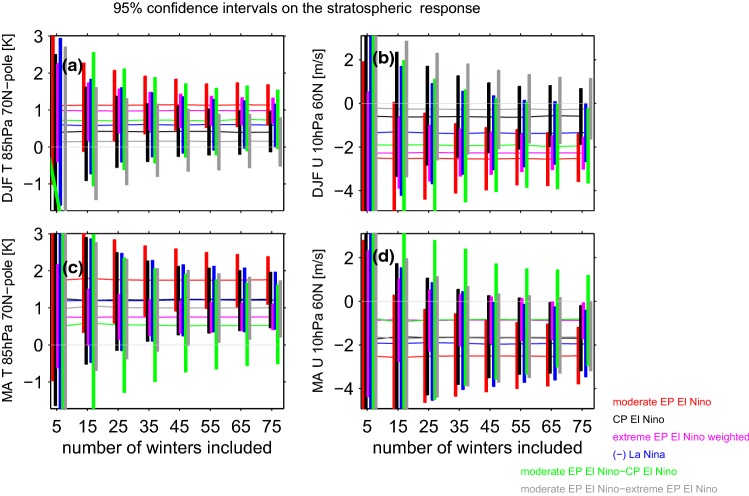
As in Fig. 3 but for the stratospheric response to ENSO **a** polar cap (70N and poleward area weighted) temperature at 85 hPa in December through February; **b** zonal mean zonal wind at 10 hPa and 60N in December through February; **c**, **d** as **a** and **b** respectively but for March and April

The Arctic stratospheric response depends linearly on the strength of the EN event during winter (Fig. 4a), as the strongest EN events lead to a proportionately stronger polar warming and vortex weakening, though during spring the ensemble mean response to the two strongest EN events falls below the linear best-fit line, and a polynomial fit is preferred. How many events are needed to robustly establish that in spring, extreme EN events may not have an impact on the Arctic stratosphere proportionate with the magnitude of SST anomalies in the tropical Pacific? Figure 5 is similar to Fig. 3 except that it focuses on the stratospheric metrics. During winter, there is no significant difference between extreme and moderate EP EN even if 75 events are considered as the gray line crosses the zero line. In contrast, during spring nonlinearities are evident if more than 45(65) events are considered in Fig. 5c (Fig. 5d): the response to moderate EP EN is larger than the response to extreme events after weighting the response in the extreme events composite by the magnitude of the underlying events.

In DJF, EP EN has a stronger effect on zonal wind at 10 hPa, 60N than CP EN, and the difference becomes statistically significant if more than 65 events are taken, consistent with Garfinkel et al. (2013); Iza and Calvo (2015) (Fig. 5c). Note that the response to CP EN as compared to climatology (black line) is insignificant in the DJF average, as the CP EN vortex response develops only in late winter (consistent with Garfinkel et al. (2013)). In spring, on the other hand, no robust difference between EP and CP EN is apparent, also consistent with Garfinkel et al. (2013).

## Changes in subpolar surface climate

**Fig. 6:**
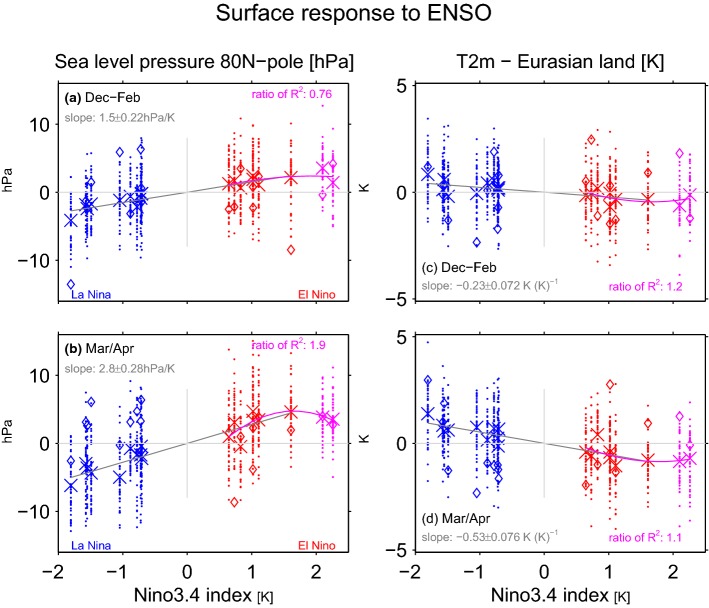
As in Fig. 4 but for the surface response to ENSO **a**, **b** sea level pressure from 80N and poleward; **c**, **d** 2 m temperatures over land areas in Eurasia area-weighted from 44N and poleward

**Fig. 7:**
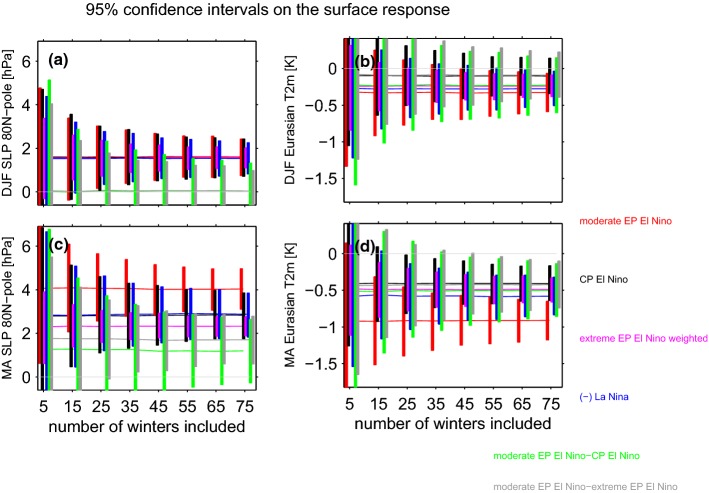
As in Fig. 3 but for **a**, **c** sea level pressure from 80N and poleward; **b**, **d** for two meter temperatures over land areas in Eurasia area-weighted from 44N and poleward

It is well established that Arctic stratospheric anomalies can propagate down to the surface (Baldwin and Dunkerton 1999; Charlton et al. 2003; Baldwin et al. 2003; Kidston et al. 2015; White et al. 2019), and we now consider the linearity of the surface impacts of ENSO in subpolar latitudes. Figure 6a, b shows the polar cap sea level pressure response to ENSO. The response to LN is equal and opposite to that of EN in both winter and spring. The response to extreme EN events is proportionately larger than the response to moderate EN events in winter though not in spring, consistent with the seasonality evident in the stratosphere. The gray line in Fig. 7c considers the robustness of this nonlinearity between extreme and moderate EN events in spring. The gray line in Fig. 7c does not touch the zero line after $\sim$ 35 events are considered so that the extreme events response is not proportionately larger than the response to moderate events. There is no difference in the response to CP EN as compared to EP EN in both midwinter and spring as the green lines cross the zero line in Fig. 7a, c, largely mirroring the stratospheric response.

One of the strongest impacts of a change in the Arctic Oscillation is cooling over Northern Eurasia (Thompson et al. 2002; Garfinkel et al. 2017; Kretschmer et al. 2018), and Figs. 6c, d and 7b, d consider the impact of ENSO on near surface temperature anomalies over land areas in Eurasia poleward of 44N. The Eurasian surface temperature response to LN is equal and opposite to that of moderate EN, but the response to strong EN events is not proportionately larger. A compositing perspective on the emergence of nonlinearities is shown on Fig. 7: During winter (Fig. 7b) the response is linear, but in spring (Fig. 7d) in contrast the gray line does not touch the zero line if more than 50 events are considered. There is a discrepancy between the two methods in DJF, as the compositing approach does not identify any nonlinearity between moderate and extreme EN but the regression approach does; however the two events included in the extreme EN composite differ as to the degree of nonlinearity, with only one featuring a response much weaker than the linear best-fit based on moderate EN events. Hence it is worth revisiting this discrepancy for future work after more extreme EN events have occurred.

EP and CP events have an indistinguishable impact on Eurasian surface temperatures in midwinter, though in spring the impact of CP events is somewhat stronger if more than 35 events are considered (i.e. the green line does not touch the zero line in Fig. 7d). This difference between CP and EP may reflect a tropospheric route for CP to affect Eurasian surface temperatures, as differences between CP and EP in spring of Arctic sea level pressure and in the stratosphere are weak.

## Frequency of SSW occurrence

Thus far we have focused on the seasonal mean response to ENSO, and we now turn our attention to changes in SSW frequency and morphology in response to ENSO. We consider two different aspects of the SSW response to ENSO: differences in morphology between EP EN and CP EN, and the frequency of SSW during LN.

### Morphology of SSW during EP EN and CP EN

Section 4 highlighted the difference in the zonal wavenumber composition of the wave flux at 100 hPa between EP EN and CP EN, and we now show that this difference has implications for the morphology of SSWs. Specifically, there are differences in the relative frequency of split versus displacement SSWs for CP EN as compared to both moderate and extreme EP EN events. The frequency of split and displacement SSWs is shown in Table 2. Displacements are clearly preferred for EP EN events, while splits are slightly enhanced in both EP EN and CP EN as compared to climatology.

**Table 2:** Frequency of splits and displacement SSWs for CP EN and EP EN events

	Frequency of SSWs	
	Split	Displacement
Climatology	0.25	0.32
Moderate EP EN	0.31	0.53
Extreme EP EN	0.26	0.50
CP EN	0.36	0.30

Displacements are clearly preferred for EP EN events, while splits are slightly enhanced in both EP EN and CP EN as compared to climatology

The binomial theorem can be used to evaluate the statistical significance of the respective changes in SSW frequency for each EN flavor, and the increase in displacement events for moderate EP EN and split events for CP EN is robust ($\mathrm{p}=9\mathrm{e}{-}5$ for displacement during moderate EP EN, and $\mathrm{p}=0.02$ for splits during CP EN). These changes in SSW morphology are consistent with the changes in the zonal composition of the wave-driving discussed in Sect. 4.

### Revisiting the La Nina-SSW relationship

As discussed in the introduction, LN does not lead to a reduction in observed SSW frequency as compared to e.g. neutral ENSO. In apparent contrast, for all metrics evaluated in this paper we were unable to detect any deviations from linearity in the seasonal mean response to LN. Is the response to LN in GEOSCCM inconsistent with the observed response to LN?

**Fig. 8:**
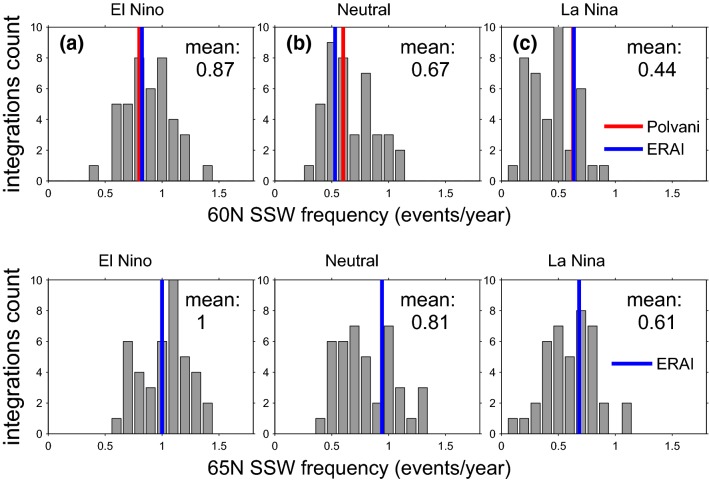
Histograms of SSW frequency across all the ensemble members during EN (left), neutral (center) and LN (right) years. Reanalysis frequencies in colors: Polvani et al. (2017) frequency in red and our reanalysis frequency in blue. The top panel defines SSWs using wind reversals at 60N, while the bottom panel defines SSWs using wind reversals at 65N

Figure 8 shows a histogram of the SSW frequency in each ENSO phase for each member of our ensemble. Figure 8a focuses on EN years, with each member in our 41 member ensemble treated separately, and for simplicity we do not differentiate between EP and CP events in this subsection. The x-axis shows the frequency of SSWs per year, and the y-axis shows how many ensemble members simulate that frequency. The colored vertical lines show reanalysis frequencies: The frequency of 0.8 events per EN year from Polvani et al. (2017), and the reanalysis frequency if we apply our ENSO classification algorithm to observations in blue at 0.78 events per EN year. We first consider the distribution of SSW frequency using zonal wind at 60N (top row). There is large variability across the ensemble, with some ensemble members indicating e.g. more than 1.2 SSW per EN winter while others indicate a frequency less than half of this. The response to LN is similarly varied (Fig. 8c), with some ensemble members indicating that LN leads to more SSW than the climatological average in GEOSCCM of 0.61 per winter (White et al. 2019), while others indicate that LN nearly shuts down SSW occurrence. Overall, however, EN leads to more SSW while LN results in fewer SSWs, as compared to neutral ENSO (Fig. 8b). However, some ensemble members have similar SSW frequency during LN and EN, i.e LN/EN SSW frequency ratio close to one. Specifically, 5 ensemble members have a LN/EN ratio of 0.9 and another ensemble member has a ratio of 1.05; hence, 14% of the ensemble members simulate SSW frequencies for EN and LN that are within 10%. Results are similar if zonal winds at 65N (bottom row) are used to define SSW events, therefore in the rest of this work we use the 60N definition only.

**Fig. 9:**
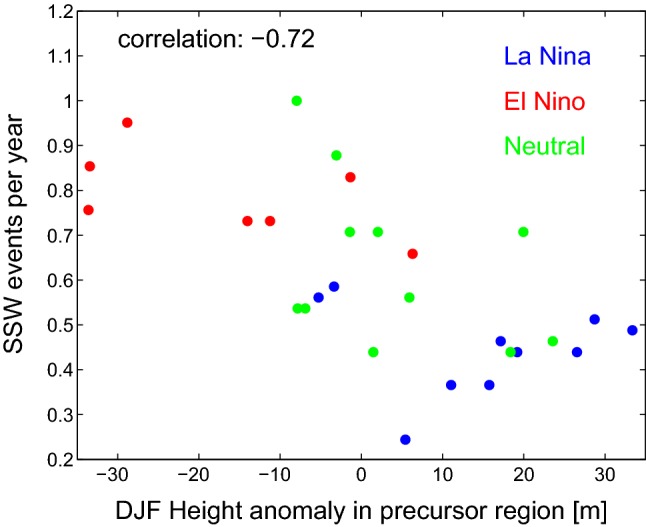
The frequency of SSWs (y axis) for each winter season averaged across all ensemble members against height anomaly at 500 hPa during each winter season (defined here as December through February) in the precursor region (x axis). This region is defined as in Garfinkel et al. (2012) and is shown with a blue box on Fig. 1. LN years are indicated with blue dots, EN years are indicated with red dots and neutral ENSO years are indicated with green dots

The relative frequencies of SSW during each ENSO phase is consistent with the North Pacific teleconnections for each ENSO phase, if one computes the ensemble mean response for each winter. As discussed in Sect. 4, an anomalously deep low in the Northwest Pacific can constructively interfere with the climatological stationary waves and lead to enhanced wave-driving. Garfinkel et al. (2012) found that a deepened low in this region is also associated with SSW events in response to ENSO. Figure 9 contrasts the 500 hPa height anomaly in the SSW precursor region (as defined by Garfinkel et al. (2012), see the blue box on Fig. 1) for each winter season averaged across all ensemble members, with the frequency of SSW events for each winter season averaged across all ensemble members. LN years are marked with blue dots, EN years with red dots and neutral ENSO years with green dots. The two metrics are clearly related ($\mathrm{r}=-0.72$), such that years with ridging in this region are associated with fewer SSW events, while troughs lead to increased SSW frequency. The correlation is highly statistically significant, and the EN winters and LN winters do not overlap in either metric.

**Fig. 10:**
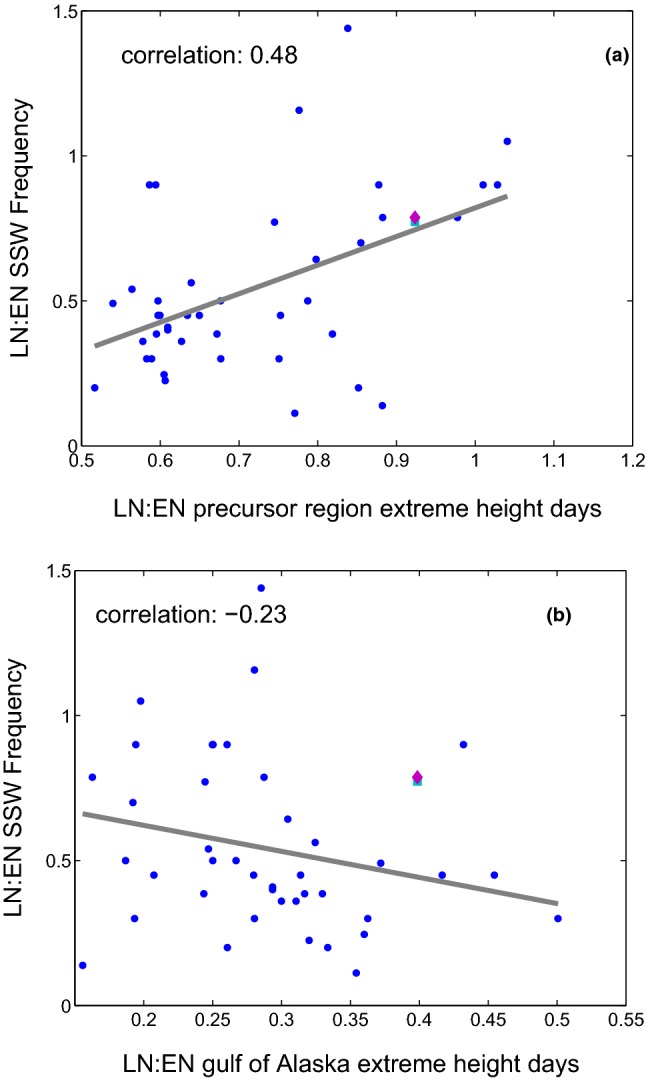
The relation between SSW frequencies and extreme height days (definition in the text) during ENSO winters during December through February in **a** the precursor region, **b** the gulf of Alaska (shown with a red box on Fig. 1). The frequency of SSWs in LN winters divided by the frequency in EN winters is shown in the y-axis. The dots denote GEOSCCM results. The square and diamond show the reanalysis result using our ENSO definition and that of Polvani et al. (2017), respectively, with the location of the markers on the x-axis based on defining ENSO seasons by the NDJF seasonal mean

**Fig. 11:**
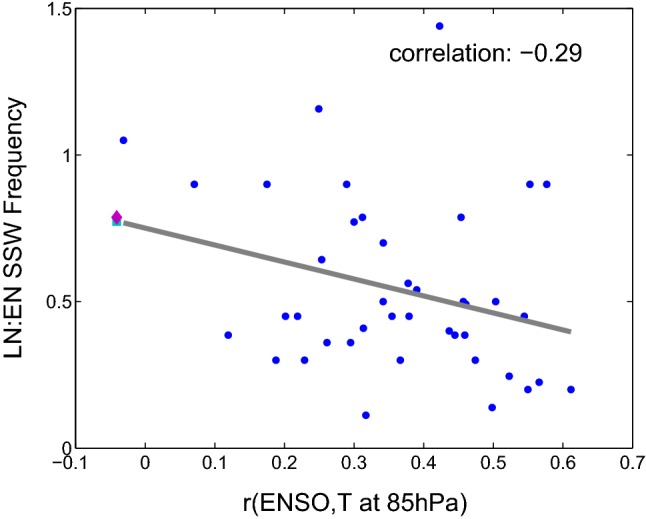
The y-axis is similar to Fig. 10. The x axis shows the correlation between Niño3.4 index and temperature anomalies at 85 hPa area weighted from 70N and poleward during December through March

In individual ensemble members, however, the frequency of SSW during EN winters and LN winters is similar, while others show almost no SSW during LN winters. What can account for the large spread in SSW frequencies across the ensemble members? We consider which aspect(s) of the ENSO teleconnection are related to the spread in the SSW response across the ensemble in Figs. 10 and 11. Figure 10a considers whether the spread in SSW frequency in our ensemble can be related to variability in the SSW precursor region. To quantify the strength of ENSO teleconnections in the SSW precursor region we compute the frequency of days in December through February in which the height anomaly at 500 hPa is more negative than $-80$ m meters (x-axis), and results are not sensitive to $\sim 30\%$ changes to this threshold. The frequency of SSWs during LN winters is divided by the frequency of SSWs for EN winters and the ratio is shown on the y-axis. Each dot represents a single ensemble member. There is a clear relationship between these parameters: in ensemble members in which the frequency of subpolar Northwest Pacific extreme lows does not depend on ENSO phase, SSW frequency also does not depend on ENSO phase. In contrast, in ensemble members in which EN leads to more frequent strong troughs in this region as compared to LN, SSW frequency is reduced during LN as compared to EN. Overall, these two metrics are significantly correlated at the $95\%$ confidence level using a Student’s test ($\mathrm{r}=0.48$).

As discussed in Garfinkel et al. (2018b), the difference in height anomalies between EN and LN peaks in the gulf of Alaska and not in the precursor region. Is there a relationship between height anomalies in the Gulf of Alaska and ENSO SSW frequency? We consider this question in Fig. 10b, which compares SSW frequencies and the strength of ENSO teleconnections on 500 hPa geopotential height in the gulf of Alaska (see the red square on Fig. 1) for each ensemble member. We see that in spite of the strong tropospheric influence of ENSO on the Gulf of Alaska, there is no relationship between the ENSO impact on the Gulf of Alaska and the ENSO impact on SSW frequency (consistent with Garfinkel et al. 2012).

We next consider the relationship between the effect of ENSO on SSWs and on seasonal mean polar stratospheric temperatures. One might expect a close relationship between the effect of ENSO on the seasonal mean stratospheric state and on SSWs, and that an ensemble member with a weaker seasonal mean stratospheric vortex during LN must necessarily have a larger frequency of SSW during LN. To examine this, Fig. 11 compares the relationship between ENSO and polar cap temperatures at 85 hPa in DJFM (x-axis) with the relationship between ENSO and SSW frequency (y-axis). We see that ensemble members with a weaker correlation between ENSO and Arctic stratospheric temperatures (i.e., r small) simulate higher LN SSW frequency as compared to EN (i.e., LN/EN larger). This relationship is not statistically significant however (correlation $-0.29$), and individual ensemble members do not necessarily show this behavior. Some ensemble members show correlations between ENSO and Arctic stratospheric temperatures in the seasonal mean that exceed 0.5 yet roughly equal EN and LN SSW frequencies. Results are similar if we compare the seasonal mean correlation of the Niño3.4 index and zonal wind anomalies at 10 hPa and 60N with the relationship between ENSO and SSW frequency: the correlation across all ensemble members is 0.33. Hence, while the seasonal mean response and SSW response are related, one should not be surprised to find periods when this connection breaks down.

## Discussion and conclusions

It is well established that the El Niño Southern Oscillation (ENSO) impacts the global atmospheric circulation in the stratosphere (Domeisen et al. 2019), and specifically El Niño leads to warmer Arctic temperatures by several Kelvin on average (Sassi et al. 2004; Garcia-Herrera et al. 2006; Garfinkel and Hartmann 2007; Camp and Tung 2007; Free and Seidel 2009).

Here we considered whether the responses to EN and LN are equal in magnitude and opposite in sign. Each EN event also differs in both the location and magnitude of maximal sea surface temperature (SST) anomalies (Capotondi et al. 2015), and it is unclear whether these differences in the SST pattern are crucial for the stratospheric response. We specifically are interested in whether the response is proportional for moderate vs extreme EN events, and to what extent the response to EN depends on the precise location of maximal temperature anomalies. The main conclusions of this study are listed in Table 3 and summarized as follows:

1. EP EN leads to enhanced wave-1 but reduced wave-2 in the lowermost stratosphere, while CP EN leads to a more moderate increase in wave-1 but not reduced wave-2 (Figs. 2, 3). This leads to a difference in the morphology of SSW forced by each ENSO flavor: EP EN have a preference for displacement events as compared to CP EN (Table 2). This difference in the zonal wavenumber composition can be linked back to the North Pacific teleconnection associated with each EN flavor, which in turn is associated with differences in the zonal wavenumber of the tropical convection (Garfinkel et al. 2018b).
2. The composited response in the Arctic stratosphere for EP EN events is stronger than for CP EN events in winter but not spring.
3. The Arctic stratospheric response and surface response to extreme EN events is somewhat weaker than one might have expected if the response was linear in the amplitude of the EN event, with deviations from linearity more pronounced in spring than in winter.
4. There is no indication of any nonlinearities in the wave driving or in the Arctic response to EN as compared to LN, and EN leads to more SSWs while LN leads to fewer SSWs in the ensemble mean (Fig. 8). In approximately $14\%$ of the ensemble members, however, there is little difference in SSW frequency between EN and LN (Fig. 10) and in some ensemble members, the SSW frequency during LN approaches 1 event per year, suggesting that a similar SSW frequency for both EN and LN can occur by chance if a relatively short sample is considered. Hence it is premature to conclude from the observational record that LN does not lead to reduced SSW frequency, due to internal atmospheric variability. That being said, intra-ensemble variability in LN SSW frequency can be related back to intra-ensemble variability in the tropospheric response to LN.

In all regions, at least 25 events in each composite are necessary before nonlinearities can be identified as statistically significant at the $95\%$ confidence level, and the nonlinearities that emerge fastest from the noise are between different flavors of EN events rather than between EN and LN. In contrast, nonlinearities in the tropospheric response were salient with far fewer events (Garfinkel et al. 2018b). Given that only approximately 20 EN events and 14 LN events are considered in the observational studies of Yu et al. (2012) and Deser et al. (2017) several of which occurred before the start of regular radiosondes throughout the Arctic in 1957, it is not surprising that it has been difficult to establish conclusively the nature of nonlinearities using observational data. Stated another way, there is substantial internal variability in the polar stratosphere that masks any true nonlinearities and leads to a small signal to noise ratio; this internal variability may also alias as apparent nonlinearities (e.g., the similar SSW frequencies during EN and LN) when none in fact may exist.

**Table 3:** A summary of nonlinearities in the response to ENSO, including the number of events that must be averaged in order for the nonlinearity to become statistically significant for a compositing approach

Summary of nonlinearities			
Region	EN vs LN	Extreme EN vs. moderate EN	EP vs CP (only composite relevant)
v’T’ 100 hPa, wave-1	Linear	Linear	$>35$ events
v’T’ 100 hPa, wave-2	Linear	Linear	$>25$ events
v’T’ 100 hPa, wave 1–3	Linear	$>65$ events for composite, linear for regression	Linear
T85 hPa, 70N-pole, DJF	Linear	Linear	Linear
T85 hPa, 70N-pole, MA	Linear	$>45$ events for composite, nonlinear for regression	Linear
U10 hPa, 60N, DJF	Linear	Linear	$>65$ events
U10 hPa, 60N, MA	Linear	$>75$ events for composite, nonlinear for regression	Linear
SLP 80N-pole, DJF	Linear	Linear for composite, linear for regression	Linear
SLP 80N-pole, MA	Linear	$>35$ for composite, nonlinear for regression	Linear
T2m Eurasia land, DJF	Linear	Linear for composite, nonlinear for regression	Linear
T2m Eurasia land, MA	Linear	$>45$ for composite, nonlinear for regression	$>35$ events

Our conclusions are based on an ensemble of GEOSCCM integrations forced with observed SSTs over the period 1980 to 2009. Such an ensemble enables an apples-to-apples comparison to the observed response to ENSO in a given season as compared to model integrations with annually repeating identical SST anomalies (as analyzed by Garfinkel et al. 2013; Rao and Ren 2016b), idealized SST patterns (Hegyi et al. 2014), or SST anomalies developed in coupled ocean-atmosphere models (Calvo et al. 2017). However we recognize three limitations of our approach:

1. The configuration used here violates energetic constraints, and does not allow for the generation of self-consistent SST anomalies and surface teleconnections.
2. SST anomalies are imposed globally, and hence it is possible that SST anomalies outside of the tropical Pacific are responsible for some of the stratospheric response. It is also reasonable to ask whether the extratropical response in our composites is the result of a single outlier included in a given composite, and is not truly representative of the other members in that composite. Furthermore, our experiments only extend for thirty years, and cannot be extended to include observed events that occurred before 1980 or since 2010. Figures 2,  4, and 9 indicate that these potential complications are not a major concern, in that the response for each member of a given composite resembles that of other members of the composite: these figures consider each ENSO event separately, and for nearly all metrics (Eurasian T2m the lone exception) shown on these figures and for both boreal winter and spring, the ensemble-mean response to events with similar SST anomalies in the Niño3.4 region indices is similar.
3. The QBO phase in our experiments is not synchronized with the observed QBO or among the experiments, and hence any nonlinear interactions between the QBO and ENSO that may exist in nature (Calvo et al. 2009; Garfinkel and Hartmann 2010) are averaged out in our results.

For nearly all metrics examined, the response to extreme EN events (e.g. 97/98) is proportionately stronger than that for moderate events in winter but not in spring, in agreement with Richter et al. (2015) and Zhou et al. (2018) in winter and with Rao and Ren (2016b) in spring (though we note that Rao and Ren (2016b) focused on midwinter). Our integrations are more similar to those of Richter et al. (2015) in that we use historical SSTs and not idealized SSTs, and it is conceivable that the specific way in which the SST anomalies in Rao and Ren (2016b) and Zhou et al. (2018) are constructed could lead to some of the discrepancies among the results of the various studies. As integrations with historical SSTs can be more easily compared to observations, they best provide the context with which to interpret nonlinearities inferred from the short observational record. Little can be concluded from the observational record due to the large amount of internal variability—cf. the scatter on Figs. 2 and 4—and the experiments examined here indicate that no such nonlinearity exists at least in winter.

The midwinter Arctic stratospheric response to CP EN events is weaker than the response to EP EN events (Fig. 5). In fact, the composited Arctic stratospheric response in winter to CP EN events is not statistically significant, and only in spring is a robust response apparent to CP EN events. These conclusions are consistent with Garfinkel et al. (2013) who imposed slightly weaker SST anomalies in their CP experiments and found a concomitantly weaker stratospheric response that developed later in the season. These conclusions are also generally similar to those of Hegyi et al. (2014) who find little difference in the stratospheric response if idealized SST anomalies of identical magnitude are placed alternately in the Central or Eastern Pacific. In contrast, the coupled ocean-atmosphere experiments of Calvo et al. (2017) indicate that CP EN has no effect on the vortex even in spring, but that study considered 43 CP EN events, and as shown in Fig. 5c, d a larger composite size is necessary before differences become robust.

The results presented in this work are all based on GEOSCCM and hence must be confirmed with other models and modeling configurations. However the model we consider simulates a realistic amount of variability over most of the key regions we identified (see Appendix B). Overall, our results suggest that it will be difficult to discern robust nonlinearities in the response to ENSO in the observational record until composite sizes grow substantially due to the smallness of the signal-to-noise ratio.

## Figures and Tables

**Fig. 12:**
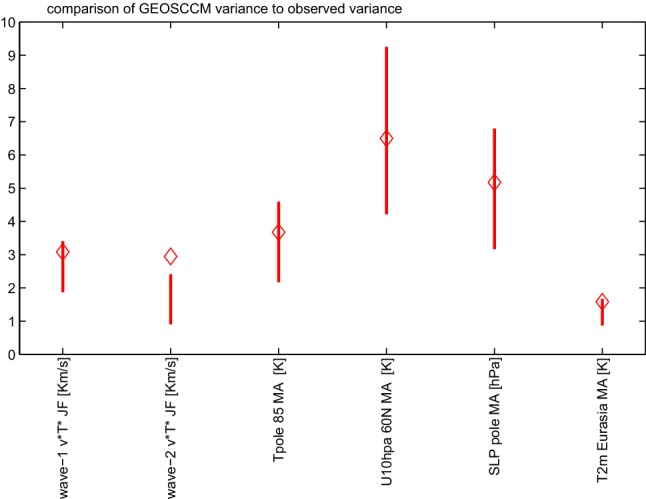
Comparison of the variance in each member of the GEOSCCM ensemble (vertical line) to the variance in reanalysis data for six key metrics discussed in the text, with the GEOSCCM range corresponding to the max and min of all ensemble members. Wave flux for wave-1 and 2 are calculated in January and February while the other four metrics are calculated in March and April

## Appendix A

Several different definitions have been used to classify winters as EN or LN, and here we explore sensitivity to the classification method used. We consider three different classification methods:

1. NDJF mean temperatures in the Niño3.4 region (as in main body).
2. The National Oceanic and Atmospheric Administration (NOAA) Climate Prediction Center definition (used by Polvani et al. 2017). Namely, if the 3-month running mean anomalies in SSTs over the Niño3.4 region exceeds either $-\;0.5$C or $+\;0.5$C for a minimum of five consecutive overlapping 3-month seasons (DJF, JFM etc.), then an El-Niño or La-Niña event is defined.
3. A new definition that better captures the dynamics behind SSWs that occur during different ENSO phases, and whose main feature is to define an ENSO season separately for SSW years and for non-SSW years. Namely, in a winter with a SSW we evaluate the mean Niño3.4 index in the two months before and the month of the SSW event, while in non SSW years we take the DJF mean. Such a definition can associate a SSW event with ENSO if the early winter Niño3.4 index was anomalous in the months preceding an SSW event, but the Niño3.4 index returned to more normal conditions later in winter. This definition explicitly ignores anomalous sea surface temperature after the SSW event.

In all cases we focus on SSTs in the Niño3.4 region in version 5 of ERSST dataset (Huang et al. 2017) with a 1980–2009 base period. Table 4 lists the years for the 3 different definitions. One can see easily that the years chosen are nearly the same for all three definitions, and hence in the main text we adopt the simplest possible definition.

**Table 4:** Years composited as EN and LN for three different ENSO definitions. Details are in Appendix A

Years classified as El Niño or La Nina for three definitions						
Year	EN			LN		
	5 months	wrt SSW	NDJF	5 months	wrt SSW	NDJF
1958	1958	1958	1958	–	–	–
1959	1959	–	–	–	–	–
1960	–	–	–	–	–	–
1961	–	–	–	–	–	–
1962	–	–	–	–	1962	1962
1963	–	–	–	–	1963	1963
1964	1964	1964	1964	–	–	–
1965	–	–	–	1965	1965	1965
1966	1966	1966	1966	–	–	–
1967	–	–	–	–	1967	1967
1968	–	–	–	1968	1968	1968
1969	1969	1969	1969	–	–	–
1970	1970	–	–	–	–	–
1971	–	–	–	1971	1971	1971
1972	–	–	–	1972	1972	1972
1973	1973	1973	1973	–	–	–
1974	–	–	–	1974	1974	1974
1975	–	–	–	1975	1975	1975
1976	–	–	–	1976	1976	1976
1977	1977	1977	1977	–	–	–
1978	1978	1978	1978	–	–	–
1979	–	–	–	–	–	–
1980	1980	–	–	–	–	–
1981	–	–	–	–	–	–
1982	–	–	–	–	–	–
1983	1983	1983	1983	–	–	–
1984	–	–	–	–	1984	1984
1985	–	–	–	1985	1985	1985
1986	–	–	–	–	–	–
1987	1987	1987	1987	–	–	–
1988	1988	1988	1988	–	–	–
1989	–	–	–	1989	1989	1989
1990	–	–	–	–	–	–
1991	–	–	–	–	–	–
1992	1992	1992	1992	–	–	–
1993	–	–	–	–	–	–
1994	–	–	–	–	–	–
1995	1995	1995	1995	–	–	–
1996	–	–	–	1996	1996	1996
1997	–	–	–	–	–	–
1998	1998	1998	1998	–	–	–
1999	–	–	–	1999	1999	1999
2000	–	–	–	2000	2000	2000
2001	–	–	–	2001	2001	2001
2002	–	–	–	–	–	–
2003	2003	2003	2003	–	–	–
2004	–	–	–	–	–	–
2005	2005	2005	2005	–	–	–
2006	–	–	–	–	2006	2006
2007	2007	2007	2007	–	–	–
2008	–	–	–	2008	2008	2008
2009	–	–	–	–	2009	2009
2010	2010	2010	2010	–	–	–
2011	–	–	–	2011	2011	2011
2012	–	–	–	2012	2012	2012
2013	–	–	–	–	–	–

## Appendix B: Variance in GEOSCCM

The main text examined the atmospheric response to different ENSO events, and paid close attention to the emergence of a robust signal from the noise of internal atmospheric variability. Specifically, there is a wide range of responses among the 41 members, and this is reflected in the wide scatter in Figs. 2,  4 and 6 and the size of the error bars in Figs.  3, 5 and 7. The large amount of internal variability in these figures makes it difficult to evaluate whether the model response to ENSO is realistic (Deser et al. 2017). However a necessary prerequisite for comparing observed and modeled ENSO teleconnections is for the model to simulate a similar amount of variance as compared to that observed, as otherwise the model does not satisfactorily capture internal atmospheric variability (Deser et al. 2017). We therefore assess whether GEOSCCM simulates a realistic amount of variance for each metric discussed in the main text in Fig. 12. We evaluate the realism of the variance by computing the variance in each region for each of the 41 ensemble members, sort the variance for the 41 members, and evaluate where the observed/reanalysis variance would lie if we were to consider it as “ensemble member 42”. The range of model variance is indicated with a vertical line on Fig. 12, and the observed variance is indicated with a diamond.

For wave-1 heat flux at 100 hPa, the variance in reanalysis data lies well within the variance simulated by GEOSCCM, and hence we expect that our conclusions with regards to the effect of ENSO in this region are relevant to nature as well. However, GEOSCCM simulates too-little wave-2 heat flux variance. Hence our conclusions with regard to wave-2 must be confirmed with other models.

GEOSCCM simulates a realistic amount of variance in the springtime polar stratosphere (though the early-winter vortex has somewhat too-little variability), in sea level pressure over the pole, and in Eurasian surface temperature (Fig. 12). Therefore, we conclude that for most metrics examined in this paper, GEOSCCM simulates a reasonable amount of internal variability.
